# Adaptor molecules mediate negative regulation of macrophage inflammatory pathways: a closer look

**DOI:** 10.3389/fimmu.2024.1355012

**Published:** 2024-02-28

**Authors:** Mirza S. Baig, Spyridoula Barmpoutsi, Shreya Bharti, Andreas Weigert, Nik Hirani, Rajat Atre, Rakhi Khabiya, Rahul Sharma, Shivmuni Sarup, Rajkumar Savai

**Affiliations:** ^1^ Department of Biosciences and Biomedical Engineering (BSBE), Indian Institute of Technology Indore (IITI), Indore, India; ^2^ Lung Microenvironmental Niche in Cancerogenesis, Institute for Lung Health (ILH), Justus Liebig University, Giessen, Germany; ^3^ Max Planck Institute for Heart and Lung Research, Member of the German Center for Lung Research (DZL), Member of the Cardio-Pulmonary Institute (CPI), Bad Nauheim, Germany; ^4^ Institute of Biochemistry I, Faculty of Medicine, Goethe University Frankfurt, Frankfurt, Germany; ^5^ Frankfurt Cancer Institute (FCI), Goethe University Frankfurt, Frankfurt, Germany; ^6^ MRC Centre for Inflammation Research, Queen’s Medical Research Institute, University of Edinburgh, Edinburgh, United Kingdom

**Keywords:** adaptor proteins, alternative activation, macrophage, inflammation, signaling mechanism

## Abstract

Macrophages play a central role in initiating, maintaining, and terminating inflammation. For that, macrophages respond to various external stimuli in changing environments through signaling pathways that are tightly regulated and interconnected. This process involves, among others, autoregulatory loops that activate and deactivate macrophages through various cytokines, stimulants, and other chemical mediators. Adaptor proteins play an indispensable role in facilitating various inflammatory signals. These proteins are dynamic and flexible modulators of immune cell signaling and act as molecular bridges between cell surface receptors and intracellular effector molecules. They are involved in regulating physiological inflammation and also contribute significantly to the development of chronic inflammatory processes. This is at least partly due to their involvement in the activation and deactivation of macrophages, leading to changes in the macrophages’ activation/phenotype. This review provides a comprehensive overview of the 20 adaptor molecules and proteins that act as negative regulators of inflammation in macrophages and effectively suppress inflammatory signaling pathways. We emphasize the functional role of adaptors in signal transduction in macrophages and their influence on the phenotypic transition of macrophages from pro-inflammatory M1-like states to anti-inflammatory M2-like phenotypes. This endeavor mainly aims at highlighting and orchestrating the intricate dynamics of adaptor molecules by elucidating the associated key roles along with respective domains and opening avenues for therapeutic and investigative purposes in clinical practice.

## 1 Introduction

Macrophages play a crucial role in the development and physiology of an organism as well as in the pathogenesis of various degenerative, infectious, and immunological diseases (1). Macrophages are a type of white blood cells that play an important role in the innate immune system. They are an important component of the first line of defense against pathogens and tumor cells by performing various functions, including ingesting and eliminating microorganisms, clearing debris and dead cells, and secreting pro-inflammatory and antimicrobial messengers. Macrophages are derived from hematopoietic stem cell-derived monocytes and embryonic yolk sac macrophages (2). They exhibit a remarkable diversity of phenotypes in different tissue environments, which is due to localized interactions with other cellular and molecular components. Macrophages actively contribute to physiological and tissue balance through a variety of cell surfaces and secreted molecules (3). To allow a functional classification of macrophage activation/phenotype, Mills and colleagues (4) introduced the terminology of M1 and M2 phenotypes, in analogy to T helper 1- and T helper 2-related inflammation, to distinguish between macrophages with pro-inflammatory and anti-inflammatory properties, respectively. M1 macrophages are often referred to as classically activated macrophages, whereas M2 macrophages are alternatively activated macrophages (5). However, we now understand that changing tissue environments provide molecular clues that lead to the emergence of a variety of macrophage phenotypes, of which the two distinct M1 and M2 subtypes appear as two possible extreme states (6, 7). In this review, we summarize 20 of the macrophage adaptor proteins that inhibit or suppress the immune response by inducing M2-like macrophages and promoting the production of anti-inflammatory cytokines.

Alternatively activated macrophages (such as M2) are usually activated by a series of stimuli (e.g., IL-4 or IL-13) and are typically observed under conditions of parasite infection and also during tissue healing and in the resolution phase of inflammation, when the burden of pathogenic infection is reduced or absent. They are characterized by their secretion of cytokines with anti-inflammatory properties. Moreover, anti-inflammatory macrophages actively support tissue remodeling and repair, e.g., by promoting angiogenesis and participating in debris clearance (8, 9). Malignant tumors attract circulating monocytes/macrophages, maturing them into tumor-associated macrophages (TAMs) with predominantly M2-like phenotypes associated with tissue remodeling and repair (10–14). Hereby, significant amounts of immunosuppressive cytokines are secreted by the anti-inflammatory TAMs, which facilitate metastasis and promote tumor growth (15).

Accurate regulation of macrophage populations is critical for proper immune function at both steady state and during disease. Deviation from this balance may result in immune pathway dysregulation (16). In the context of cellular signaling pathways, adaptor proteins exert a critical influence on the modulation of signal transduction. Despite their lack of inherent enzymatic activity, adaptor proteins are able to transmit signals to desired targets *via* other molecules using their characteristic domain structures (17). Adaptor proteins are equipped with a variety of functional domains that enable specific interactions between proteins and between proteins and lipids (18–23). The modular structure of adaptor proteins, which includes one or more specific domains that enable their interaction with various other proteins, is a characteristic feature shared by all adaptor proteins (17). Adaptor molecules play a key role in the core of various receptor-mediated signaling pathways and act as important mediators bridging the gap between receptors and other molecular components (24).

Recent therapeutic strategies to combat macrophage-mediated inflammation include signal modulation to enable a transition from a pro-inflammatory state to an anti-inflammatory state (25). Hereby, adapter proteins can critically influence the outcome of an external signal, either activating or inhibiting receptor-induced signal transduction (17). Macrophages receive a plethora of microenvironmental stimuli (cytokines, chemokines, and growth factors) that bind on surface receptors and initiate intracellular signaling and that need to be integrated by, among others, adaptor proteins (26). Activating adaptor proteins include myeloid differentiation 88 (MyD88), Toll/interleukin-1 receptor (TIR) domain-containing adaptor protein (TIRAP), TIR domain-containing adaptor-inducing interferon-β (TRIF), TNF receptor-associated factor 6 (TRAF6), growth factor receptor-bound protein 2 (Grb2), and caspase recruitment domain-containing protein 9 (CARD9). They regulate the cellular response to a stimulus by inducing the formation of the appropriate signaling complex, spatiotemporal regulation of signaling, activation of binding components, kinase regulation, and sequestration of specific proteins. The amplification of signaling and cell activation is determined by the recruited proteins, complex localization, and signal duration. Also, the binding of the adaptor protein to its target can be sufficient for its activation (27). Signaling pathways that are regulated by the activating adaptor proteins include the NF-κB pathway, AP-1, MAPK, IRFs, JAK/STAT, and PI3K/Akt (28, 29). The final effect of the activation of the aforementioned signaling pathways in macrophages is the production of pro-inflammatory cytokines, cytoskeleton rearrangement, regulation of apoptosis, and proliferation (30).

In contrast to activating adaptor proteins, inhibitory adaptor proteins serve the vital role of negatively regulating signal transduction. Based on the domains that constitute their structure (TIR, ligase domain, SH2, SH3, and IRF association domain), they exhibit different mechanisms of signal inhibition. For example, they can directly or indirectly induce the ubiquitination and subsequent degradation of Toll-like receptors (TLRs) or the activating adaptor proteins, resulting in the inhibition of signal transduction (31, 32). By these means, they may be instrumental in switching macrophage phenotypes from a pro-inflammatory state toward an anti-inflammatory state. In this review, we describe 20 of these inhibitory adaptor proteins such as Sterile α- and Armadillo motif-containing protein (SARM), Toll-interacting protein (TOLLIP), Src-like adaptor protein (SLAP), DNAX-activating protein of 12 kDa (DAP12), *Astragalus* polysaccharide (APS), LnK, β-arrestin, suppressor of cytokine signaling-1 (SOCS-1), interleukin-1 receptor associated kinase-M (IRAK-M), downstream of kinase 3 (DOK-3), interferon regulatory factor 4 (IRF4), interferon regulatory factor 7 (IRF7), G protein-coupled receptor 108 (GPR108), NOD-like receptor family caspase recruitment domain family domain containing 5 (NLRC5), Disabled-2 (DAB2), Triad-3A, cytoplasmic linker protein 170 (CLIP170), interleukin-1 receptor-associated kinase 1/4 (IRAK-1/4), adaptor protein c-Cbl-associated protein (CAP), and Src kinase-associated phosphoprotein 2 (SKAP2). We explore their role in regulating macrophage activation.

## 2 Sterile α- and Armadillo motif-containing protein

**Figure 1:**
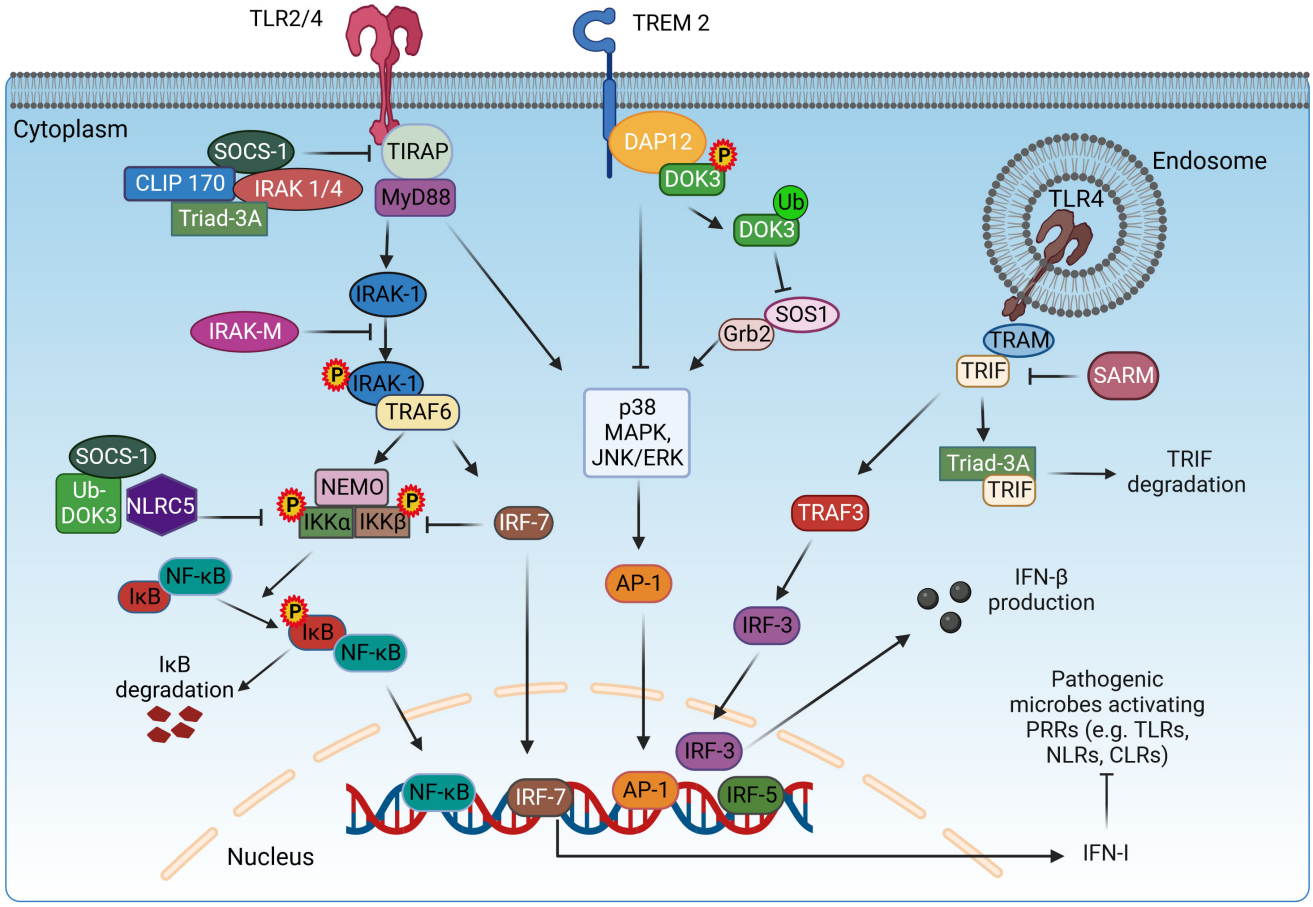
Adaptor proteins mediating the signaling of TLR receptors. TLR signaling is mediated *via* two pathways: MyD88-dependent and TRIF-dependent pathway. In the MyD88-dependent pathway, TIRAP mediates the interaction of MyD88 with TLR2/4 for the initiation of the signaling cascade. The adaptor proteins SOCS-1, IRAK1/4, CLIP170, and Triad3A inhibit the function of TIRAP. MyD88 recruits IRAK-1, which will be phosphorylated and bind to TRAF6. Once activated, TRAF6 acts as an E3 ubiquitin ligase, ubiquitinates, and activates NEMO, IKKα, and IKKβ to induce the phosphorylation of IκB and subsequent dissociation from NF-κB, resulting in the nuclear translocation of NF-κB. SOCS-1, Ub-DOK-3, NLRC5, and IRF7 inhibit the activation of IKKα/β, downregulating the NF-κB pathway. In parallel, MyD88 induces the p38, MAPK, and JNK/ERK signaling for the activation and translocation of AP-1 in the nucleus. In the TRIF-dependent pathway, TRIF indirectly interacts with TLR4 *via* TRAM and activates IRF3 *via* TRAF3, which translocates in the nucleus and induces the expression of IFN-β. The role of TRIF is inhibited by SARM. DAP12 associates with TREM2 upon stimulation and induces the phosphorylation and translocation of DOK-3 on the cell membrane, resulting in the inhibition of MAPK and ERK pathways. Meanwhile, ubiquitin-mediated degradation of DOK-3 leads to SOS1 degradation and inhibition of ERK pathway. TLR2/4, Toll-like receptor 2/4; TIRAP, TIR domain-containing adaptor protein; MyD88, myeloid differentiation 88; IRAK-1/4/M, interleukin-1 receptor-associated kinase 1/4/M; TRAF3/6, TNF receptor associated factor 3/6; NEMO, nuclear factor-κB essential modulator; IKKα/β, inhibitory kappa B kinase α/β; NF-κB, nuclear factor-κB; SOCS-1, suppressor of cytokine signaling-1; CLIP170, cytoplasmic linker protein 170; NLRC5, NOD-like receptor family caspase recruitment domain family domain containing 5; DOK-3, downstream of kinase 3; DAP12, DNAX-activating protein of 12 kDa; Grb2, growth factor receptor-bound protein 2; SOS1, Son of sevenless homolog 1; TRIF, TIR domain-containing adaptor-inducing interferon-β; TRAM, TRIF-related adaptor molecule; SARM, Sterile α and Armadillo motif-containing protein; DAB2, Disabled-2; IRF3/5/7, interferon regulatory factor 3/5/7; AP-1, activator protein 1; IFN-β, interferon-β; NLRs, nucleotide-binding oligomerization domain (NOD)–leucine-rich repeat (LRR)-containing receptors; CLRs, C-type lectin receptors. Created with BioRender.com.

The adaptor protein SARM is a TLR adaptor protein identified in 2001 by Mink et al. The 690 amino acid long SARM adaptor protein is encoded by the SARM gene located on chromosome 17q11 (33) and is highly conserved in *Caenorhabditis elegans*, mice, and *Drosophila* (34). SARM consists of three domains: an Armadillo repeat motif (ARM) at the N-terminus, two sterile alpha motifs (SAMs), and a TIR domain at the C-terminus (35). The SAM domain is involved in protein–protein interactions through homo- and heterotypic oligomerization to an octamer (36). The 40 amino acid ARM domain mediates autoinhibition as well as interaction with other proteins and beta-catenin with its ligands (35). The TIR domain is responsible for interaction with TLRs and mediates the innate immune response (37). Of the five TIR domain adaptor proteins of TLR, SARM has a unique function by negatively regulating the immune response (35). SARM inhibits the signaling pathway mediated by TLR3 and TLR4 and thus the downstream activation of NF-κB, IRF3, and activator protein-1 (AP-1) (38) through direct TIR–TIR interaction with TRIF and MyD88 (39) (**Figure 1**). The glycine residue (G601) in the BB loop of the SARM-TIR domain is essential for interaction with the MyD88 adaptor protein (39). Moreover, in rheumatoid arthritis, there is a negative correlation between SARM and TLR2-induced IL-1β expression, and higher SARM levels result in an enhanced response to anti-TNF-α therapy (40). SARM is not exclusively a negative regulator of inflammation and has also been shown to selectively promote TLR4- and TLR7-induced CCL5 expression in macrophages (41). However, SARM regulates TLRs, and TLRs can also control the expression of SARM. For example, treatment of RAW264.7 macrophages with TLR2 ligands increases SARM expression, an effect that requires TLR9 (42). SARM is involved in numerous cellular processes and pathologies, including neuropathy (43), apoptosis, antiviral immune responses (44), mitophagy, and neuronal death (38). SARM’s regulatory role in macrophages can be ambiguous at times. Functioning as messengers within cells, these molecules facilitate communication among various proteins. The complexity and specificity of these molecules contribute to the uncertainty in their roles, as they may have diverse functions depending on the cellular context and the molecules they interact with. Additionally, SARM molecules can act as both facilitators and inhibitors in controlling macrophage behavior. Their functionality is dynamic, adapting to ongoing cellular events. Consequently, the apparent ambiguity in their role in macrophage regulation arises from these intricate and context-dependent interactions.

## 3 DNAX-activating protein of 12 kDa

The signaling adaptor molecule DAP12, also known as killer cell activating receptor-associated protein (KARAP) or tyrosine kinase binding protein (TYROBP), was originally discovered for natural killer (NK) cells (45–47), and later studies showed that it also plays a role in macrophages, dendritic cells, and monocytes (48–50). DAP12 consists of an extracellular domain, a transmembrane domain, and an intracellular domain that specifically includes an immunoreceptor tyrosine-based activation motif (ITAM) (51). The interaction of DAP12 with the receptor present on the cell surface is due to the presence of an aspartic acid residue in its transmembrane domain that forms an electrostatic association (51, 52). The binding of the ligand to the DAP12-bound receptor activates SRC family kinases and, in turn, leads to phosphorylation and activation of the ITAM tyrosine of DAP12 (53). The phosphorylated tyrosine of ITAM serves as a docking site for several tyrosine kinases, namely, ZAP70 and Syk, which further downstream signal transduction (54). Blocking the binding of DAP12 to Syk mediated by Ocilrp2 reduces lipopolysaccharide (LPS)-induced IL-6 production (55, 56). Signaling pathways such as FcεRIγ and CD3ζ, which are considered to be carriers of downstream DAP12 signaling, have been extensively studied (57, 58). One study showed that the knockdown of DAP12 gene in microglial BV2 cells resulted in an increase in mRNA levels of pro-inflammatory cytokines in response to LPS by stabilizing TREM2 (59). TREM2–DAP12 interaction inhibits the activation of Ras and ERK through the recruitment of the proteins Dok-3, Grb2, and Sos1, therefore inhibiting the TLR4-induced pro-inflammatory cytokine production (60) (**Figure 1**). DAP12/TREM2 signaling was also found to inhibit macrophage activation against non-glycosylated mycolic acid mycobacteria (61). Knockdown of DAP12 has been shown to increase the production of pro-inflammatory cytokines in alveolar macrophages during porcine reproductive and respiratory syndrome virus (PRRSV) arterivirus infection (62). When DAP12 was reintroduced into DAP12−/− macrophages, production of the pro-inflammatory cytokine TNF was significantly reduced (63). IL-4 is thought to be present in alternatively activated macrophages (M2), and thus, its regulation in response to DAP12 is a topic of interest. RNA silencing targeting DAP12 in human monocyte-derived macrophages significantly decreased IL-4-induced macrophage fusion (64). Macrophage fusion refers to the formation of multinucleated giant cells, which play a role in the immune response in granulomatous diseases. It has been suggested that signaling through a receptor such as TLRs or FcγRIII, which tend to activate the ERK pathway, may be inhibited by DAP12 (63). Therefore, further studies focusing on DAP12 are needed to investigate its precise mechanistic role in macrophage polarization.

## 4 Suppressor of cytokine signaling-1

SOCS-1 was discovered in 1997 (65) and referred to by several terms: SOCS-1, STAT-induced signal transducer and activator of transcription (STAT) inhibitor-1 (SSI1), structure and function of a new STAT-induced STAT inhibitor, and JAK binding protein (JAB) (66). In humans, SOCS-1 protein consists of 211 amino acids, while in rats and mice, it has 212 amino acids. The SOCS-1 protein consists of a central SH2 domain flanked by an amino terminal with a 12 amino acid kinase inhibitory domain/region and a carboxy terminal with an approximately 40 amino acid SOCS box. The KIR domain is critical for inhibition of the JAK2 kinase domain, the SH2 domain binds to phosphorylated tyrosine regions, and the SOCS box mediates ubiquitin-based proteasomal degradation through the formation of a functional E3 ligase enzyme (67). Originally, SOCS-1 was identified as a negative regulator of cytokine (68) and interferon signaling (69) by interfering with JAK/STAT signaling. SOCS-1 inhibits the JAK/STAT signaling pathway in three ways: the SH2 domain inhibits the kinase activity of JAK by binding to the kinase domain of JAK, binding of the phosphotyrosine residues of the cytokine receptor, and ubiquitination and degradation of activated JAK by the elongin-BC complex (67, 70). SOCS-1 interacts with IRAK-1 and NF-κB, promotes their degradation, and negatively regulates the MyD88-dependent TLR signaling pathway (**Figure 1**). SOCS-1 also regulates the LPS-induced TLR signaling pathway by acting on the MyD88-dependent and MyD88-independent signaling cascade (71) and by interacting with phosphorylated TIRAP, leading to its ubiquitination and further degradation. SOCS-1 inhibits phosphorylation of p65 by TIRAP and further activation of NF-κB (72). TRIF/TRAM mediates the MyD88-independent pathway leading to the activation of NF-κB and interferon regulatory factor 3 (IRF3). IRF3 also activates interferon (IFN)-β, which activates STAT-1 *via* signaling through the IFN-α/β receptor. This is an indirect mechanism that inhibits TLR signaling *via* inhibition of the JAK/STAT signaling pathway induced by IFN-β (73). Overexpression of SOCS-1 reduces tyrosine phosphorylation, a step critical for activation of STAT-1 (74, 75).

In activated M2 macrophages, there is increased expression of SOCS-1 but not SOCS-3, and SOCS-1 polarizes macrophages toward an M2 phenotype (76, 77). Expression of SOCS-1 in macrophages inhibits palmitic acid- and LPS-induced signaling and protects mice from insulin resistance and systemic inflammation (78). Survival in IFN-γ−/− SOCS-1−/− or IFN-γ−/− SOCS-1+/− mice was reduced compared to that in wild mice. In SOCS-1−/− macrophages, there was increased production of TNF-α and NO and decreased endotoxin tolerance in response to LPS due to increased phosphorylation of I-κB and p38. NF-κB expression was decreased in macrophages overexpressing SOCS-1 (79). These studies suggest that SOCS-1 regulates LPS-stimulated macrophages independently of IFN-γ (79, 80).

SOCS-1 expression is remarkably high in the early stages of inflammation and decreases in later stages due to methylation of the SOCS-1 promoter region by DNA methyltransferase 1 (DNMT1), which silences SOCS-1 gene expression in LPS-treated crude macrophages. DNMT1 may be a potential target for inhibition of macrophage activation. Apart from inflammation, SOCS-1 also plays a role in cancer (81). Suppression of SOCS-1 can enhance antitumor immunity or promote tumor-promoting inflammation, depending on the cell type (82). Using the CRISPR-Cas9 method, the IRF1–SOCS-1 axis was found to inhibit CXCL9 expression and STAT1 signaling, thereby limiting antitumor immunity (83). However, the knockdown of the SOCS-1 gene in macrophages enhances anticancer inflammation and reduces tumor development (82).

## 5 Interleukin-1 receptor-associated kinase-M

IRAK-M belongs to the IRAK family but is known to lack kinase activity (84). IRAK-M is also popularly known as IRAK3 and has been studied in the negative regulation of inflammatory states associated with TLRs (**Figure 1**) (85, 86). IRAK-M is contrarian in function compared to the other members of the family. IRAK-M includes an N-terminal death domain (DD), a kinase domain (KD), and a C-terminal domain (CTD) that contains a conserved motif that helps bind to TRAF6. IRAK-M interacts with other proteins in the family through its DD. The lack of an aspartate residue in its active site has been postulated as the main reason for the lack of kinase activity (85, 87).

Its negative role in inflammatory signaling has been highlighted in several studies. In IRAK-M−/− mouse macrophages, the levels of pro-inflammatory cytokines IL-6, IL-12 p40, and TNF-α were found to increase significantly in response to LPS treatment compared to wild-type (WT) macrophages. Similarly, the effects of IRAK-M deficiency were also evident in the increased signaling *via* NF-κB, JNK, p38, and ERK in LPS-stimulated macrophages (86). TGF-β signaling is known to be involved in the expression of IRAK-M, as evidenced by detailed studies in human peripheral blood mononuclear cells (PBMCs) and mouse macrophage cell lines (88). Downregulation of M2 macrophage surface marker expression was clearly demonstrated under IRAK-M knockdown conditions (89). Regarding the role of IRAK-M in cancer, studies show that tumor cells induce the expression of IRAK-M on human monocytes *via* CD44 and TLR4, resulting in monocyte deactivation and decreased expression of pro-inflammatory cytokines (90). In a lung cancer mouse model, IRAK-M (−/−) mice injected with Lewis lung carcinoma cells exhibited reduced tumor growth compared with WT mice, whereas tumor-associated macrophages isolated from these mice expressed a stronger M1 phenotype. TGF-β signaling was found to promote IRAK-M expression in macrophages during lung tumor growth (88).

## 6 Downstream of kinase 3

DOK-3 belongs to the Dok family, which includes a total of seven members (Dok-1–7). DOK-3 is known to be involved in the regulation of tyrosine kinase-related signaling (91). Expression of Dok-3 is mainly observed in B cells, plasma cells, neutrophils, macrophages, and dendritic cells (92–95). Structural analysis revealed the presence of a C-terminal domain (190 amino acids), a central domain, and an N-terminal pleckstrin homology (PH) domain. Sequence analysis further revealed that among the three domains, the C-terminal domain varies, while apparent homology with Dok-3 and Dok-2 was found between the central and N-terminal domains (96). Dok-3 plays an important role as a scaffold in inflammatory processes because it lacks enzymatic activity. In neutrophil granulocytes, Dok-3 was found to suppress CARD9 signaling during fungal infection mediated *via* CLR (95). Dok-3 has been shown to regulate TLR4–ERK-mediated inflammatory response in response to LPS mediation and is also involved in DAP12-mediated inhibition of LPS-stimulated inflammatory signaling in macrophages (**Figure 1**). Dok-3 KO mice had higher mortality and serum TNF-α levels compared with WT mice exposed to LPS (94). Another study reported that NF-κB activation and production of pro-inflammatory cytokines IL-1β, TNF-α, and IL-6 were negatively correlated with Dok-3 upon LPS stimulation (97). In gliomas, it was shown that higher expression of DOK-3 strongly correlates with M2 macrophage markers and higher macrophage infiltration (98). Another research showed that vitamin 6 treatment of LPS-stimulated macrophages decreases the expression of pro-inflammatory cytokines, and this effect was abolished in DOK-3 KO macrophages but was enhanced upon overexpression of DOK-3 (99). Also, CpG-mediated ubiquitination and subsequent degradation of DOK-3 *via* interaction with TRAF6 leads to increased production of IL-6 and TNF-α in macrophages (100).

## 7 Interferon regulatory factor 7

The IRF family of mammals consists of nine members: IRF1–IRF9 (101, 102). IRF7 was originally discovered in the context of Epstein–Barr virus (EBV) infection and has since evolved to become a central controller of type I IFNs in response to pathogenic infections. It is triggered by activating signaling cascades initiated by pathogen recognition receptors (PRRs) that identify pathogenic genetic material. Abnormal formation of type I IFNs has been associated with a variety of diseases, such as malignancies and autoimmune diseases. Therefore, precise control of IRF7 expression and function is critical for the proper production of type I IFNs to maintain normal physiological functions mediated by IFNs. As shown by the process of phosphorylation, which serves as a clear indicator of its activation, post-translational modifications play a crucial role in controlling the activity of IRF7 (103). Activation of IRF7 is a prerequisite for its function as a transcription factor (103). In its resting state, inactive IRF7 is localized in the cytoplasm until activated. In response to pathogenic infection, phosphorylation of IRF7 is triggered, leading to its translocation from the cytoplasm to the nucleus. In the nucleus, IRF7 forms a transcriptional complex with other co-activators that binds to the promoter regions of specific genes and initiates their transcription (102) (**Figure 1**).

Intriguingly, the IRF family and the NF-κB family have co-evolved and share common evolutionary features. These families are both activated by signaling pathways originating from identical PRRs and IκB kinase (IKK). They also cooperate synergistically in regulating important cytokines such as IFN-β and together serve as central components in innate immune responses (104). The human IRF7 gene is located on chromosome 11p15.5 and is responsible for the production of four unique isoforms: IRF7A, IRF7B, IRF7C, and IRF7D. This discovery highlights the gene’s remarkable ability to generate multiple variations, each with its own characteristic features. The human IRF7A (503 amino acids, 55 kDa) differs from its mouse counterpart, IRF7 (457 amino acids, 52 kDa). Notably, IRF7 and its closest relative, IRF3, play a critical role in regulating the type I interferon (IFN-α/β) response (102).

IRF7, a critical transcription factor, has significant regulatory power over the transition from M1 to M2 phenotype, a vital process. Moreover, in pathological interventions, induction of IRF7 expression by IFN-β1 proves to be an effective intervention that effectively attenuates the pro-inflammatory response of microglial cells after injury. By *in vivo* manipulation, activation of IRF7 expression in microglial cells after spinal cord injury resulted in a profound reduction in pro-inflammatory behavior, demonstrating its potent effect in attenuating inflammatory responses (105). IRF7 emerges as a central factor essential for triggering robust induction of type I IFN genes when TLR7 or TLR9 is stimulated. Through phosphorylation, TBK1 modifies IRF7 and converts it to its active form, enabling its participation in important cellular processes and promoting the production of IFN-responsive genes (102). For example, lidocaine inhibits H1N1 virus replication in macrophages by upregulating IFN-a4 *via* TBK1-IRF7 and JNK-AP1 signaling pathways (106). When primary macrophages were transduced with the active form of IRF7, the production of type I IFNs and their tumor-killing effect were increased (107). The potential explanation for IRF7’s ability to exhibit different functions is linked to the specific contexts in which macrophages operate (tissue microenvironment), whether they involve the activation or inhibition of specific inflammatory pathways, which provides special power to IRF7 to exhibit transition (switching mechanism) from the profound M1 to the distinguished M2 phenotypic expression, making it the most critical transcription factor used in therapeutics. The roles may be ambiguous and influenced by the particular *in vivo* conditions, and the specific downstream targets of IRF7 can influence the overall macrophage phenotype.

## 8 NOD-like receptor family caspase recruitment domain family domain containing 5

NLRC5 belongs to the family of intracellular PRRs and NOD-like receptors (NLRs) (108). Immune cells such as monocytes, macrophages, and lymphocytes are known to express NLRC5 at high levels (109). These proteins contain a nucleotide-binding site (NBS) consisting of leucine-rich repeats (LRRs). Pathogens are recognized by the C-terminal domain of these protein molecules. Several domains such as the caspase recruitment domain (CARD), a pyrin domain (PYD), and the baculovirus inhibitor repeat domain (BIRD) together form the N-terminus (109, 110). Oligomerization and activation of NLRs depend on the central nucleotide-binding oligomerization domain. The localization of NLRC5 depends on its expression level; i.e., at elevated expression, it is located in the cytoplasm, whereas at physiological expression levels, it is present in the nucleus (109).

The protective role of NLRC5 in angiogenesis and intimal hyperplasia has been well studied (111, 112). It was observed that NLRC5 was able to downregulate NF-κB signaling in macrophages in coordination with heat shock protein 8 (HSPA8). In the absence of NLRC5, levels of IL-6 were found to increase in macrophages, leading to activation of cardiac fibroblasts (113). The effect of NLRC5 on the production of pro-inflammatory cytokines was also demonstrated in another study, in which overexpression of NLRC5 on macrophages downregulated the expression of IL-6 and TNF-α, whereas its suppression had the opposite effect. The JAK2/STAT3 pathway was shown to control the expression levels of NLRC5 (114). Several studies have shown that IFN-γ regulates the gene expression of NLRC5 upon infection (115, 116), but how NLCRC5 affects RIG-I and IFN response is still controversial. Priya et al. reported that through interaction domain mapping, NLRC5 interacts with RIG-I *via* its N-terminal DD and that NLRC5 enhances antiviral activity in a leucine-rich repeat domain-independent manner. This finding identifies a novel role for NLRC5 in RIG-I-mediated antiviral host responses against influenza virus infection, distinguished from the role of NLRC5 in MHC class I gene regulation (117). It was also shown that NLRC5 acts as a mediator of the IFN-mediated antiviral signaling pathway and that overexpression of NLRC5 activates the IFN-specific response and upregulates antiviral genes (116). In contrast, another study showed that NLRC5 inhibits the NF-kB signaling pathway and negatively regulates the type I IFN signaling *via* interaction with the RIG-I and MDA5 (118). However, despite its effect on type I IFN signaling and RIG-I, the antiviral effect of NLRC5 can be supported by its role in promoting the activation of NLRP3 inflammasome. In addition to that, NLRC5 overexpression leads to increased activation of caspase-1, which converts pro-1L-1β to the active 1L-1β (119). Moreover, a recently published study demonstrates a novel NLRC5-mediated antiviral pathway for the inhibition of dengue virus infection. In this pathway, the antiviral effect of NLRC5 is exerted *via* the interaction of NLRC5 with the viral non-structural protein 3 (NS3) protease domain, followed by the ubiquitination of NS3 protease domain and degradation of NS3 through a ubiquitin-dependent selective macroautophagy/autophagy pathway (120). Overall, different pathways can mediate the role of NLRC5 in antiviral immune response, which still needs further investigation. In *Helicobacter*-modulated gastric inflammation and lymphoid formation, NLRC5 has been shown to function as a negative regulator. It was observed that significantly higher levels of cytokines and chemokines were produced in NLRC5−/− THP-1 macrophages under the influence of *Helicobacter pylori* than in WT THP-1 macrophages (121).

## 9 Disabled-2

DAB2 is a clathrin- and cargo-binding endocytic adaptor protein known for its multiple functions in signaling pathways regulating cellular migration, tumor suppression, and other important homeostatic biological activities. DAB2 may help promote immunological tolerance and reduce inflammatory responses (122). This can be validated by several studies. In myeloid cells, the absence of Dab2 promotes an inflammatory phenotype. Systemic inflammation was increased in Dab2-deficient bone marrow, as evidenced by higher serum levels of IL-6 and expression of inflammatory cytokines in the liver (123). Dab2 expression was found to be increased in M2 macrophages but decreased in M1 macrophages, and genetic deletion of Dab2 caused macrophages to develop a pro-inflammatory M1 phenotype (124). Deletion of Dab2 increased activation of TRIF-dependent interferon regulatory factor 3 and production of interferon-inducible genes and subsets of inflammatory cytokines (125) such as IL-12 and IL-6 (126). Dab2 acts as a negative immunological regulator of TLR4 endocytosis and signaling, suggesting a unique role for a Dab2-associated regulatory circuit in modulating macrophage inflammatory responses (125). In contrast, Dab2 has been shown to be involved in the activation of macrophages to the M1 phenotype during central nervous system (CNS) inflammation. It is associated with early activation of macrophages and astrogliosis during CNS inflammation (127). Dab2 promotes central nervous system inflammation by possibly increasing the expression of reactive oxygen species (ROS) in macrophages and microglia (128). One possible reason for Dab2’s diverse roles could be that it interacts with various partners in distinct signaling pathways at different body regions. For example, in the CNS, it promotes M1 polarization, but in the liver, it promotes M2 polarization by reducing inflammatory cytokines. Thus, understanding the molecular signaling of Dab2 can be used for therapeutic purposes in various types of diseases.

## 10 Triad-3A

Triad3A is an E3 ubiquitin-protein ligase of the RING finger type that regulates macrophage activity *via* the mediation of TLR signals (129). Activation of macrophages toward the regulatory M2 type and prevention of their conversion to the M1 phenotype are its key roles (130). Triad3A has been shown to bind to and degrade the TLR4 adaptor proteins TIRAP, TRIF, and RIP1, limiting the release of pro-inflammatory cytokines (**Figure 1**). It was also discovered that Triad3A triggers K48-linked ubiquitination and degradation of TLR4 and TLR9, preventing the production of pro-inflammatory cytokines (129). The effect of Triad3A-mediated degradation of TLR4 and TLR9 by ubiquitination has also been studied in heart disease, where it was found to play a protective role in the development of cardiac hypertrophy and may improve cardiac function (131). In contrast, K48-linked ubiquitination and degradation of TLR4 triggered by Triad3A have a negative effect on mitochondrial bioenergetics and disease pathology in a model of diabetic cardiomyopathy (132).

In addition, it was discovered that inflammatory cytokine synthesis and necroptosis are limited by Triad3A-dependent necrosomal degradation (133). Through ubiquitin-mediated degradation of the tumor necrosis factor receptor-associated factor 3 (TRAF3) adaptor, Triad3A also has a negative effect on the RIG-I RNA-sensing pathway. Triad3A expression also inhibited the expression of type 1 interferon and antiviral genes by phosphorylating IRF3 and blocking its activation, thereby reducing inflammation (134). In addition to inflammation, Triad3A was also discovered to play a role in autophagy. In RAW264.7 and bone marrow-derived macrophages activated with LPS, it was also discovered that Triad3A interacts with and degrades Beclin-1 through ubiquitination, blocking TLR4-mediated autophagy (135). Thus, we could cure a number of diseases by using it in a variety of ways, such as targeting siRNA to disrupt Triad3A and limiting bacterial growth by triggering autophagy (136).

## 11 Cytoplasmic linker protein 170

CLIP170, an adaptor protein that controls the dynamics of the growing plus end of microtubules (MTs), consists of two conserved glycine-rich (CAP-Gly) domains of the cytoskeleton-associated protein and two tandem repeats of zinc knuckle motifs. It was found that CLIP-170 is a critical regulator of the stabilization of MT and that stabilized MTs play an important role in cell phagocytosis in activated macrophages (137). Actin polymerization events critical for phagocytosis are controlled by CLIP-170 *via* regulation of the recruitment of the actin core-forming protein form in mDia1 (138). It was discovered to play a role in modulating the anti-inflammatory form of macrophage activation. In one study, TLR4 signaling was found to be negatively regulated by CLIP170 by targeting TIRAP (**Figure 1**). Silencing of CLIP170 enhanced LPS-induced production of pro-inflammatory cytokines, while overexpression of CLIP170 in mouse macrophages decreased the expression of pro-inflammatory cytokines, indicating its anti-inflammatory role (139). Pregnenolone hormone was also found to stimulate CLIP170-mediated ubiquitination, leading to increased degradation of TIRAP and TLR4 inhibition (140). In a separate study on the anti-inflammatory properties of SOCS-3, it was discovered that SOCS-3 interacts with the MT plus-end binding proteins CLIP-170 and CLASP2 *via* its N-terminal domain, resulting in the SOCS-3–CLIP-170/CLASP2 complex, which has anti-inflammatory properties (141). Therefore, through various studies, it has been discovered that CLIP170 plays an important role in polarizing macrophages toward the anti-inflammatory type and can be used for therapeutic purposes in a variety of chronic diseases.

## 12 Interleukin-1 receptor-associated kinase 1/4

IRAK1 and IRAK4 are threonine/serine kinases. The N-terminal death domains of MyD88, IRAK4, and IRAK2 help form a multimeric spiral signaling complex (myddosomes) (142, 143). Asymmetric trans-autophosphorylation of IRAK4 dimers recruited to the myddosome leads to the recruitment of IRAK1, which subsequently participates in extensive autophosphorylation and detaches from the myddosome (142, 144). TLR4/2 signaling is negatively regulated by IRAK1/4, which expresses an auto-active IRAK4 that causes TIRAP degradation (145) (**Figure 1**). This suggests a regulatory or anti-inflammatory effect of IRAK1/4, which consequently reduces the M1 phenotype. Several studies support its anti-inflammatory role. First, LPS-induced sepsis was alleviated by specific inhibition of IRAK1 (146). In another study, IRAK and Rip2 were found to be deregulated in sarcoidosis (147). Inhibition or silencing of IRAK1/4 reduced Ox-LDL-induced CD36 expression, thereby reducing the development of macrophage foam cells involved in the release of pro-inflammatory cytokines (148). In the same context of macrophage-derived foam cells, Ox-LDL inhibits LPS-induced expression of IFN-β by activation of IRAK1/4. Activated IRAK1/4 induces the mono-ubiquitination of TANK, which in turn will inhibit the recruitment of TBK1 to TRAF3 and the activation of IRF3 (149). LPS-induced formation of ROS was reduced by inhibiting IRAK1/4, which is located downstream of TLR (150). Thus, depending on the type of disease and condition, IRAK1/4 can be used for therapeutic purposes.

## 13 *Astragalus* polysaccharide

APS (formerly known as adaptor protein with PH and SH2 domains (SH2B2)) is a member of the Src homology 2 B (SH2B) family, which includes three members (SH2B1, SH2B2, and SH2B3). It exhibits a conserved configuration consisting of an initial dimerization domain (DD), a central pleckstrin homology domain (PH), and a terminal Src homology 2 (SH2) domain, all of which contribute to its structural integrity (151). The three components of the SH2B family were originally identified as signaling substances involved in immune cell stimulation (152–154). APS is a macromolecular substance obtained from *Astragalus membranaceus*, which consists of complex polysaccharides rich in α-(1 4) glycosidic bonds, with key constituents like glucan, glucose, galactose, and arabinose influencing its structure and function (155). APS exhibits a variety of properties, especially immune-enhancing, anti-inflammatory, and antioxidant properties (156–158), and is considered an immunostimulant to enhance human immune response (155, 156).

Studies have revealed that APS acts as a potent bio-immunomodulator, augmenting both non-specific and specific immune responses including a role as a vaccine adjuvant (159). *In vitro* experiments showed that APS prevented the conversion of LPS-stimulated THP-1 macrophages to the M1 phenotype. This effect was accompanied by a marked reduction in the formation of ROS and pro-inflammatory mediators (TNF-α, IL-6, and IL-12) and a phenotypic conversion toward M2 polarization, accompanied by the release of anti-inflammatory factors (IL-4, IL-10, and Arg-1) (160). According to Zhou et al., APS enhances the immunomodulatory properties of RAW 264.7 macrophages by activating the TLR4 and MyD88 signaling pathways (161). Further research suggests that APS functions as an immune receptor that specifically targets TLR4 in both mouse macrophages and B cells (162). APS suppresses TNF-α and IL-1β production by preventing NF-κB activation and attenuating phosphorylation of the ERK and JNK signaling pathways (163).

In addition, APS treatment showed a beneficial effect on endothelial cell proliferation while attenuating apoptosis. These results could be replicated *in vivo*, demonstrating the remarkable potential of APS in alleviating thoracic aortic complications in diabetic rats (160). Moreover, SH2B2 connects with the insulin receptor, promotes robust activation of the insulin signaling pathway, and supports its optimal functionality (164). In cell cultures, SH2B2 shows a compelling ability to bind to JAK2 *via* its SH2 domain, resulting in a strong enhancement of JAK2 activation (165). This mechanism contributes to the regulation of energy balance and body weight, particularly by affecting leptin and growth hormone signaling (165, 166).

## 14 Toll-interacting protein

TOLLIP is a universally expressed protein discovered in 2000 (167). It plays a prominent role as an adaptation molecule in the innate immune response *via* modulation of IL-1RI- and TLR2/4-mediated signaling pathways (168). The receptors IL-1R and TLRs have homologous cytosolic TIR domains that activate signaling pathways upon stimulation with IL-1 and LPS (169). Tollip was originally recognized as a mediator in IL-1 signaling. It has since been found to interact directly with TLR2 and TLR4 (170) and reduce inflammation (171). Structurally, Tollip has three domains with distinct functions and binding partners: the Tom1-binding domain (TBD) at the N-terminal, which is connected to the coupling of ubiquitin to the ER-degradation domain (CUE) through the conserved 2 domain (C2) consisting of 130 amino acids (172). Of these three domains, the CUE domain plays a critical role in inhibiting the IL-1RI/TLR4 signaling pathways by interacting with the TIR domains of cell surface receptors and also with IRAK-1 and IRAK-2 proteins, thereby inhibiting autophosphorylation (173). Amino acid sequence 1–53 encodes TBD, which is responsible for interaction with clathrin, ubiquitin, and TOM1 during early endosomal interactions (174). The 130 amino acid C2 domain binds to phospholipids, preferably phosphoinositides, allowing Tollip to localize to cell membranes (175, 176).

**Figure 2:**
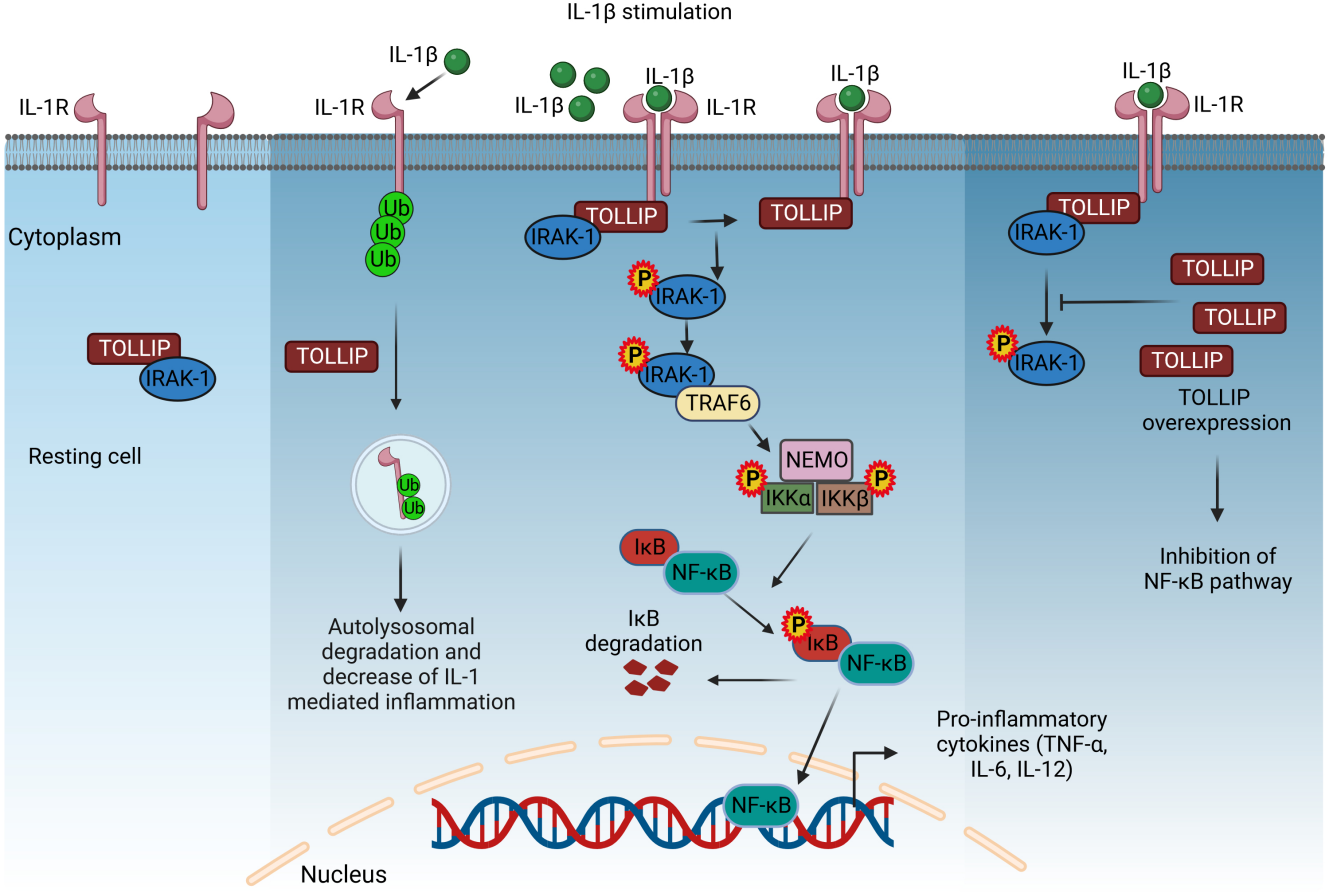
TOLLIP-mediated regulation of IL-1R signaling. In resting cells, TOLLIP forms a complex with IRAK-1, and upon stimulation with IL-1β, this complex translocates and binds to the TIR cytoplasmic domain of IL-1R. Co-recruited adaptor proteins induce the phosphorylation of IRAK-1, dissociation from the complex, and binding to TRAF6, which activates the NF-κB signaling, as described in **Figure 1**. TOLLIP decreases IL-1R-induced inflammation by promoting the endosomal degradation of ubiquitinated IL-1R. Overexpression of TOLLIP inhibits the NF-κB pathway and the expression of pro-inflammatory cytokines by inhibiting the phosphorylation of IRAK-1. IL-1β, interleukin 1β; TOLLIP, Toll-interacting protein; IRAK-1, interleukin-1 receptor-associated kinase 1; TRAF6, TNF receptor-associated factor 6; NEMO, nuclear factor-κB essential modulator; IKKα/β, inhibitory kappa B kinase α/β; NF-κB, nuclear factor-κB. Created with BioRender.com.

In resting cells, Tollip is present in a complex with interleukin-1 receptor-associated kinase 1 (IRAK-1), and upon stimulation with IL-1β, the complex recruits to the cytoplasmic TIR domain of IL-1R *via* its accessory proteins. However, phosphorylation of IRAK by co-recruited adaptor molecules leads to complex breakdown. Phosphorylated IRAK is also associated with TRAF6, leading to activation of the NF-κB pathway (177). However, overexpression of Tollip leads to inhibition of NF-κB-mediated release of pro-inflammatory cytokines by inhibiting phosphorylation of IRAK1 (167). In addition, Tollip decreases IL-1-induced inflammation by causing endolysosomal degradation of ubiquitinated IL-1R1 (31) (**Figure 2**). Tollip also plays a critical role in the cyclic guanosine monophosphate adenosine monophosphate synthase stimulator of interferon genes (cGAS–STING) and acts as a stabilizer of STING, preventing its activation and thus that of the transcription factors IRF3 and NF-κB (178).

In inflammatory bowel disease (IBD), Tollip overexpression in peritoneal macrophages inhibits LPS-induced production of pro-inflammatory cytokines and increases anti-inflammatory cytokines, demonstrating the role of Tollip in macrophage polarization (179). A resveratrol derivative, RM, which is being studied for the treatment of inflammatory diseases, downregulates LPS-induced pro-inflammatory cytokine production, an effect mediated by increasing Tollip expression (180). In essence, Tollip is a negative regulator of acute inflammation. However, at a low dose of LPS, it translocates from lysosomes to mitochondria, increasing ROS levels and causing chronic inflammation (181). In addition to its important role in inflammation, Tollip is also involved in vacuolar trafficking, autophagy, and nuclear interactions (182).

## 15 β-Arrestin

β-Arrestins 1 and 2 are proteins that are widely distributed throughout the body and affect the signaling of G protein-coupled receptors (GPCRs). β-Arrestin 2 performs a critical function as a signaling adaptor and scaffold protein in modulating cellular inflammatory responses (183). Studies have linked β-arrestins to the signaling and activation of TLRs and genes (184–186). These adaptor proteins, β-arrestins 1 and 2, play a critical role in modulating the function of heterotrimeric guanine nucleotide-binding regulatory (G) proteins by forming complexes with GPCRs (186). Moreover, they are characterized as cytosolic proteins that facilitate the process of desensitization and internalization of activated G protein-coupled receptors (187, 188).

In addition, recent findings have demonstrated the role of β-arrestins 1 and 2 as scaffold/adaptor proteins in the activation of various MAPKs (183, 189). These include extracellular signal-regulated kinase 1/2 (ERK 1/2), c-Jun N-terminal kinase (JNK), p38 kinases, and Src family kinases in the context of GPCR signaling. β-Arrestin 2 functions as a scaffold for several MAPK components, including JNK and ERK (190, 191). This function promotes phosphorylation, activation, and spatial concentration of MAPKs in specific cellular compartments, leading to their accumulation (191). MAPKs serve as important mediators in the signaling pathways of TLRs and play a central role in the transmission and transduction of TLR signals. MAPKs serve as vital intermediaries in the signaling pathways of TLRs, playing a pivotal role in transmitting and relaying the TLR signals (185). In addition to their involvement in the regulation of MAP kinases, studies have shown that β-arrestins also play a role in modulating the activity of NF-κB (184, 192, 193). Studies have provided evidence for the role of β-arrestin 2 in TLR signaling (183, 194). Specifically, studies have shown that overexpression of β-arrestins 1 and 2 in HEK cells stably expressing TLR4 leads to attenuation of NF-κB activation induced by LPS (186, 194).

In the absence of β-arrestin 2 (β-arrestin 2(−/−) mice), LPS administration resulted in increased expression of pro-inflammatory cytokines in bone marrow-derived macrophages (BMDMs). Moreover, LPS-induced mortality was increased in galactosamine-sensitized mice lacking β-arrestin 2. These comprehensive observations provide compelling evidence that β-arrestins serve as indispensable negative modulators of innate immune activation *via* TLRs (195). Another interesting study demonstrates that the production of IL-6 in polymorphonuclear leukocytes (PMNs) stimulated by LPS was significantly increased in β-arrestin 2 (−/−) mice compared to their wild-type counterparts (+/+). Therefore, β-arrestin 2 serves as an inhibitory regulator of pro-inflammatory mediator production in PMNs (196). Moreover, the absence of β-arrestin 2 negatively affects the formation of IL-10 in response to LPS stimulation (185).

In a remarkable study, researchers demonstrated a direct interaction between β-arrestin and TRAF6 in the activation of TLRs or IL-1 receptors. This interaction serves to prevent autoubiquitination of TRAF6 and thus inhibit subsequent activation of NF-κB and the AP-1 signaling pathway (197). Moreover, β-arrestin 2 shows the ability to directly interact with IκB-α, thereby effectively preventing the phosphorylation and degradation of IκB-α. Consequently, this interaction effectively inhibits the activation of NF-κB (194). In addition to their above functions, both β-arrestin 1 and β-arrestin 2 play a role in cardiac function, as they are expressed in cardiac tissue and have been associated with the regulation of normal cardiac function. In the infarcted heart, a remarkable upregulation of β-arrestin 2 expression was found specifically in infiltrated macrophages, where it exerts a suppressive effect on inflammatory responses. Interestingly, mice lacking arrestin 2 (KO) show a higher mortality rate after myocardial infarction than wild-type mice. Furthermore, the absence of arrestin 2 (KO) in mice resulted in increased production of inflammatory cytokines. These results provide compelling evidence that infiltrated macrophages in β-arrestin 2 KO mice elicit an exaggerated inflammatory response in the infarct region (187).

Upon binding of an agonist to GPCRs, a group of enzymes known as GRKs, comprising seven homologs, phosphorylates intracellular threonine or serine residues of GPCRs. This phosphorylation process enables the recruitment of β-arrestins to agonist-activated GPCRs (198). Due to their ability to prevent G protein activation by steric hindrance and their interaction with clathrin and adaptin, β-arrestins contribute to the desensitization and internalization of GPCRs *via* clathrin-coated pits (199). Apart from their involvement in regulating GPCRs, recent studies have shed light on the ability of β-arrestin to function as a signal transducer (198, 199). As a result, β-arrestins have emerged as promising targets for potential therapeutic interventions in cardiovascular diseases such as heart failure (200, 201).

## 16 LnK

**Figure 3:**
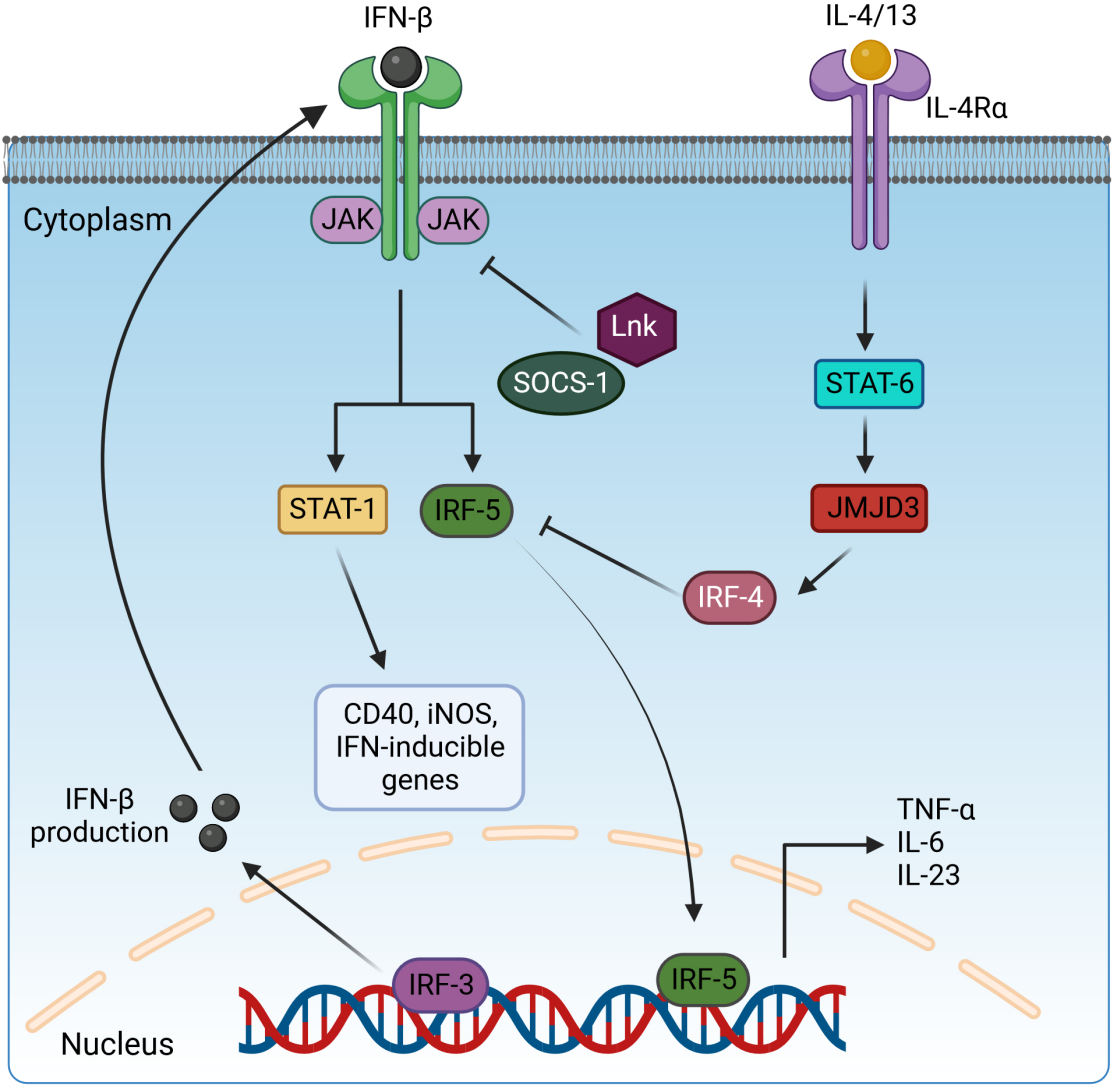
Regulation of pro-inflammatory cytokine expression by interleukin receptors *via* STAT and IRF transcription factors. Activated IRF3 translocates in the nucleus and induces the expression IFN-β. IFN-β is secreted and binds to interferon receptors and activates the JAK/STAT1 signaling to drive the expression of CD40, iNOS, and IFN-inducible genes and IRF5 for pro-inflammatory cytokine production. However, this pathway is negatively regulated by the inhibitory adaptor proteins LnK and SOCS-1, which interfere with STAT1 activation, and IRF4, which blocks IRF5 signaling. IRF4 is induced *via* the IL-4/13/STAT6/JMJD3 cascade. IFN-β, interferon β; JAK, Janus kinase; STAT-1/6, signal transducer and activator of transcription-1/6; IL-4/13/6/23, interleukin-4/13/6/23; IRF3/4/5, interferon regulatory factor 3/4/5; SOCS-1, suppressor of cytokine signaling-1; JMJD3, Jumonji domain-containing protein-3; CD40, cluster of differentiation 4; iNOS, inducible nitric oxide synthase. Created with BioRender.com.

LnK (SH2B3) belongs to the SH2B family of adaptor proteins, along with SH2B1 and SH2B2 (202). This family shares a common structural framework characterized by proline-rich regions, pleckstrin homology (PH), SH2 domains (202, 203) responsible for binding phosphotyrosine in target proteins, and an N-terminal dimerization domain with a phenylalanine zipper pattern (204). This pattern facilitates the formation of homo- and hetero-dimers among SH2B family members (204). LnK plays a central role as a dynamic adaptor protein in modulating a variety of signaling pathways orchestrated by Janus kinases (JAKs) (**Figure 3**) and receptor tyrosine kinases (RTKs) (202, 203). The involvement of adaptor proteins that bind to both RTKs and JAKs is of great importance in controlling the intricate network of cytokine signaling pathways (205). Without their own enzymatic activity, these adaptor proteins assume the central role of molecular platforms that skillfully orchestrate and harmonize a variety of signaling events (206). The SH2B family, which includes members of the Src homology 2 proteins, emerges as a dynamic group of adaptor proteins involved in a diverse array of signaling pathways. In mice lacking SH2B family members, the absence of LnK results in the most striking abnormalities in hematopoiesis, highlighting its profound importance in this biological process (205, 206). Another study also indicated the crucial role of LnK (SH2B3) in maintaining normal hematopoiesis as a key negative regulator of cytokine signaling (204).

**Figure 4:**
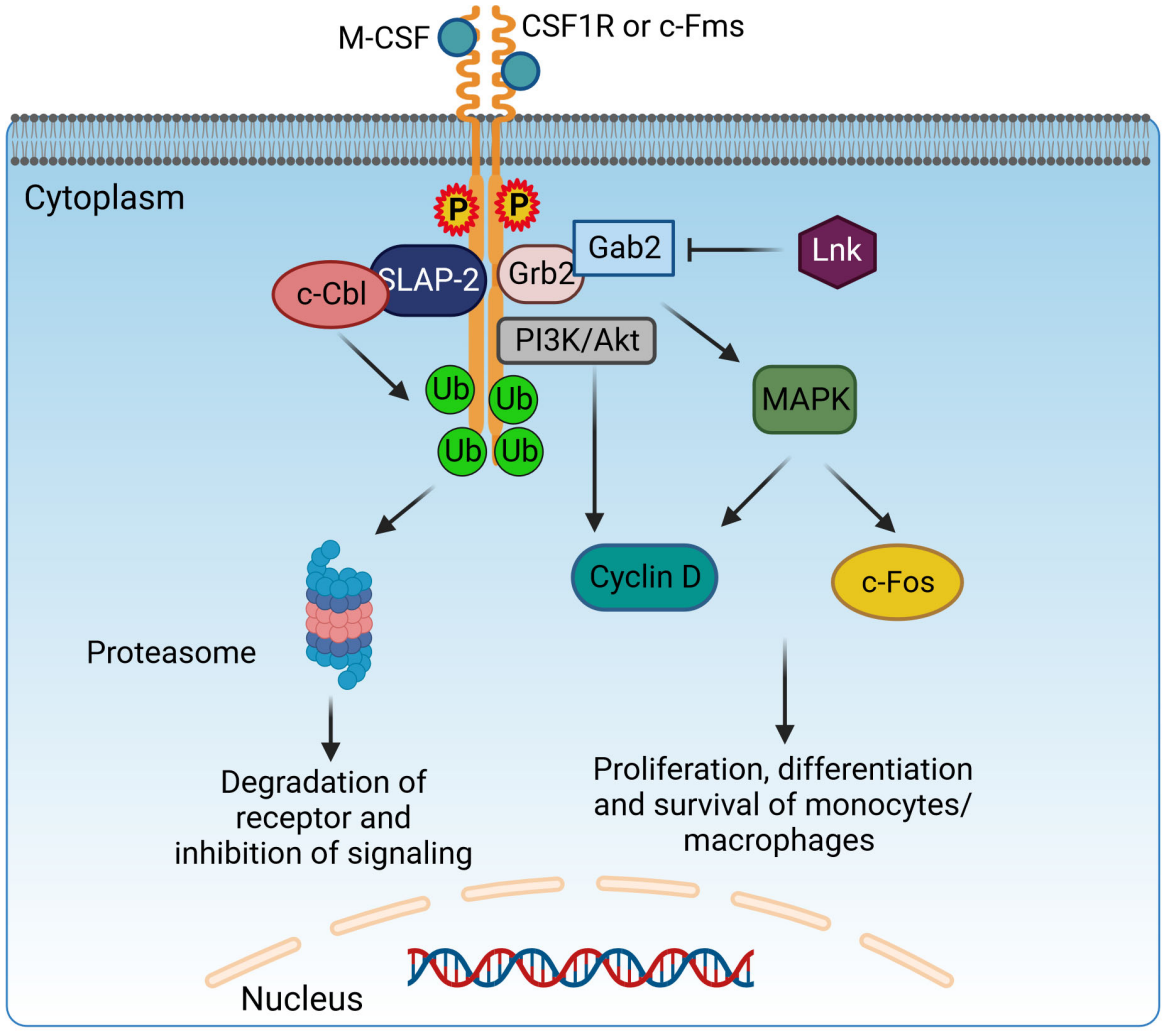
Adaptor protein SLAP-2 regulates CSF1R-mediated differentiation and survival of macrophages. Binding of M-CSF to CSF1R (c-Fms) leads to dimerization and tyrosine phosphorylation of CSF1R, generating docking sites to recruit adaptor molecules possessing SH2 domains, like Grb2. Grb2 binds with Gab2 to activate PI3K/Akt and MAPK signaling and induce the proliferation, differentiation, and survival of monocytes/macrophages *via* Cyclin D and c-Fos. Lnk has an inhibitory effect on this pathway. However, SLAP2 can bind both c-Fms and c-Cbl and induce c-Cbl-dependent ubiquitination, internalization, and degradation of the receptor, therefore inhibiting the CSF1R signaling pathway. CSF1R, colony-stimulating factor-1 receptor; c-Fms, colony-stimulating factor-1 receptor; SLAP2, Src-like adaptor protein 2; Grb2, growth factor receptor-bound protein 2; Gab2, GRB2-associated-binding protein 2; MAPK, mitogen-activated protein kinases. Created with BioRender.com.

Lnk forms a direct physical association with c-Fms, effectively attenuating its activity, which includes regulation of macrophage progenitor cell proliferation, macrophage colony-stimulating factor (M-CSF)-induced migration, and production of ROS (**Figure 4**). The M-CSFR (c-Fms) contributes in complex ways to the control of macrophage proliferation, differentiation, and survival, exerting a critical influence on the regulation of various macrophage functions. Upon interaction with the M-CSF ligand, c-Fms undergoes tyrosine phosphorylation, resulting in docking sites for molecules with SH2 domains. Phosphorylation of Akt was enhanced and sustained in mouse macrophages with LnK knockout in response to M-CSF, whereas phosphorylation of ERK was significantly attenuated. Moreover, Lnk effectively suppressed the migration response of macrophages triggered by M-CSF. After stimulation with zymosan, LnK knockout mouse macrophages showed a remarkable increase in the production of ROS in an M-CSF-dependent manner, highlighting the central role of Lnk in regulating the formation of ROS in response to zymosan-induced activation. Lastly, the absence of LnK led to significant alterations in the cytokine production of macrophages, emphasizing the critical role of Lnk in modulating the immune response and influencing the release of inflammatory cytokines by these vital immune cells (207). LnK plays a crucial role as a negative modulator of TNF signaling, effectively attenuating the pro-inflammatory phenotype (203). Overall, manipulating the expression levels of LnK in macrophages offers a distinctive therapeutic strategy to enhance the innate host defenses, representing a novel approach for potential clinical intervention (207).

Lnk expression levels also correlate with metabolic diseases. Obesity has been shown to lead to a decrease in Lnk expression in immune cells. Deficiency of LnK triggers adipose tissue inflammation and disrupts glucose tolerance, indicating its central role in maintaining the balance of adipose tissue inflammation and ensuring optimal glucose regulation. Through comprehensive qPCR analysis, investigators examined the expression of inflammatory cytokines in adipose tissue and found a significant increase in the levels of IL-1β, IL-12, TNF, and IFN-γ in LnK−/− mice, highlighting the central role of LnK in modulating the inflammatory cytokine profile in adipose tissue (208). Moreover, investigations have demonstrated that Lnk-deficient mice display impaired glucose tolerance and diminished insulin responses, highlighting the critical involvement of LnK in governing these essential metabolic functions (208). The inhibitory effect of the SH2 domain is widely recognized, and its efficacy can be abrogated by the specific point mutation R392E, highlighting the critical importance of this mutation (207). A specific genetic variation in the LnK/SH2B3 gene, known as a missense variant, has been linked to an increased risk of several autoimmune diseases, including diabetes. LnK plays a central role in regulating the ability of dendritic cells (DCs) to modulate the fate of Th1 or regulatory T cells (Treg) in response to stimulation by granulocyte-macrophage colony-stimulating factor (GM-CSF) and IL-15, underscoring its essential function in orchestrating this complex immune response (209).

## 17 Interferon regulatory factor 4

IRFs are intracellular proteins that play a critical role in macrophage polarization and immune cell maturation (210, 211). The IRF family consists of nine members, of which IRF1, IRF5, and IRF8 drive the macrophage into the M1 phenotype, and IRF3 and IRF4 switch the macrophage into the anti-inflammatory M2 phenotype (210). IRF4 is a 450 amino acid long transcription factor consisting of a conserved 115 amino acid long DNA-binding domain (DBD) at the N-terminal and IRF association domains (IADs) at the C-terminal (212). The IAD is also associated with an autoinhibitory region at the C-terminus that binds to the DBD and regulates its interaction with DNA (212, 213). The IAD is connected to the DBD *via* a flexible linker. The DBD forms a helix-loop-helix motif with the help of the conserved five tryptophans, which mediates binding to DNA by recognizing GAAA and AANNNGAA sequences (210). The IAD helps interact with other interferons and transcription factors (212).

IRF4 functions as an endogenous antagonist of TLR signaling and competes with IRF5 for binding to the adaptor protein MyD88, suppressing the M1 polarization of macrophages (214, 215). Overexpression of IRF4 increases the release of the anti-inflammatory cytokines IL-4 and IL-10 (216), and in the same way, M2 marker genes (Arg1, Ym1, and Fizz1) are decreased in IRF4 deficiency (217). IRF4-deficient mice are more susceptible to LPS-induced inflammation with increased release of pro-inflammatory cytokines such as TNF-α and IL-6. In IRF4-deficient macrophages, NF-κB and JNK pathway-based cytokine production was increased after stimulation with LPS (218). During helminth infection, the Jumonji domain containing-3 (Jmjd3) methylates IRF4, a step required for its induction (217). Jmjd3 is regulated by STAT6 signaling induced by IL-4. Thus, M2 macrophage polarization is controlled by STAT6-Jmjd3-IRF4 signaling (**Figure 3**) (219). Another publication indicates that the Jmjd3-IRF4 axis plays a role in M2 polarization induced by IL-13 through the NOTCH4 pathway (220).

## 18 Src-like adaptor protein

SLAP has a similar structure to Src Homology2 (SH2) and Src Homology3 (SH3) and contains Grb2, Nck, and Crk domains. However, unlike the Src family, SLAP lacks a catalytic tyrosine kinase domain (221). The SH3–SH2 domain facilitates protein–protein interactions by recognizing phosphorylated tyrosine- or proline-rich sequences. The SH3–SH2 junction sequence is shorter in SLAP/SLAP2 than in Src kinases, resulting in a closer association of Src domains through continuous β-sheet formation (222). The SH2–SH3 domains are flanked on one side by amino terminals and on the other side by unique carboxyl terminals. The myristoylated N-terminus helps associate with membranes, while the non-myristoylated N-terminus isoform is localized in the nucleus (223). The most studied SLAP proteins are SLAP1 and SLAP2, both of which differ in size; they have a difference of 15 amino acid residues. SLAP1 has 276 amino acid residues, while SLAP2 contains 261 amino acids (223, 224). SLAP acts mainly as a negative regulator on intracellular signaling pathways in macrophages as well as in B (222) and T lymphocytes (223, 225, 226). SLAP interacts with cell surface receptors, recruiting a ubiquitin machinery that leads to receptor degradation, thereby inhibiting signal transduction (221). SLAP2 genes are expressed in BMDMs (227). SLAP2 regulates signal transduction of a tyrosine kinase receptor, colony-stimulating factor-1 receptor (CSF-1R) (221, 228). The ligand for CSF-1R is colony-stimulating factor-1 (CSF1), which regulates macrophage growth and differentiation (229). The binding of CSF1 to the tyrosine kinase receptor CSF-1R leads to its dimerization and phosphorylates tyrosine residues, further mediating downstream signaling *via* the recruitment of SH2 adaptor protein and PTB proteins (230). c-Cbl downregulates CSFR-1 signaling. The SH2 domain of c-Cbl binds to an activated tyrosine kinase, and the RING finger domain has ubiquitin ligase activity, leading to ubiquitination and eventual degradation of the receptor (231) (**Figure 4**). In BMDMs, the association of SLAP2, c-Cblans, and CSF-1R receptor is necessary for the inhibition of downstream signaling. SLAP2 binds with c-Cbl *via* its unique carboxy-terminal tail (227). In FD-Fms cells, the dominant-negative SLAP2 mutant inhibits the binding of c-Cbl to the receptor, thereby inhibiting receptor ubiquitination, internalization, and degradation. SLAP2 plays a role in the recruitment of c-Cbl to activated CSF-1 receptors and the consequent downregulation of CSF-1R signaling by promoting the internalization and degradation of activated receptors (228).

## 19 G protein-coupled receptor 108

Sophisticated organisms have evolutionarily evolved their intrinsic and acquired defense mechanisms, enabling them to combat foreign substances and pathogens with maximum efficiency. GPR108, a member of the seven-transmembrane protein (7TM) domain family, exerts potent stimulation of NF-κB signaling when overexpressed. Remarkably, its role in a physiological setting proves to be antagonistic to signaling pathways initiated by TLRs (232). Originally identified as lung 7TM receptor-1 and receptor-2 (LUSTR1 and LUSTR2), these genes have since been renamed GPR107 and GPR108, respectively, by the HUGO Gene Nomenclature Committee. The human GPR107 gene with 18 exons is located at locus 9q34.2-3 and covers an area of 86.4 kb. The corresponding cDNA encodes a protein with 552 amino acid residues. In contrast, the mouse GPR108 gene, which is closely related but lacks homology, contains 17 exons and is located in region 17C-D, spanning a shorter length of 12.8 kb (233). The most closely related mammalian ortholog, GPR107, has 49% similarity in the amino acid sequence compared to GPR108 in mice (232).

GPR108 is considered one of the major candidates among candidate genes involved in innate immunity, further highlighting its significant role in this important defense mechanism (234). One possible mechanism is the interplay of GPR108 and TLR4 mediating the interaction with MyD88, modulating the E3 ligase TRAF6 to facilitate ubiquitination of MyD88. Of note, the immunostimulatory function of GPR108 is limited by the expression of TIRAP. According to Donget et al., cells from mice lacking GPR108 show increased secretion of cytokines and enhanced activation of NF-κB and IRF3 signaling pathways. Conversely, GPR108-deficient macrophages, in which GPR108 is restored, show decreased signaling responses. Joint expression of TLRs and GPR108 results in decreased activation of the NF-κB and IFN-β promoter, in contrast to expression of TLRs or GPR108 alone. Upon activation of TLRs, the amount of GPR108 increases, resulting in its interaction with TLRs and their antagonists suppressing the expression of MyD88 and hindering its binding to TLR4 by blocking the ubiquitination of MyD88 and GPR108 negatively regulating MyD88. Of note, the antagonistic effect on GPR108 is exerted by TIRAP, an adaptor protein essential for TLR and MyD88 signaling (232).

## 20 c-Cbl-associated protein

CAP or c-Cbl-associated protein, also known as sorbin and SH3 domain containing (Sorbs1), belongs to the sorbin homology (SoHo) family of adaptor and scaffold proteins. It is abundantly expressed in immune system cells as well as in cardiac tissue, adipose tissue, and skeletal muscle. CAP plays an important role in regulating cell adhesion, migration, cytoskeletal element reorganization, membrane trafficking, and intracellular signal transduction (235, 236). It also has a protective antiviral function in coxsackievirus virus B3 (CVB3)-induced myocarditis (235). CAP promotes type I IFN production while limiting the release of cytotoxic cytokines, thereby setting a balanced and non-harmful antiviral response. In a recent study by Vdovenko et al., it was shown that the expression of pro-inflammatory cytokines in mouse fibroblasts, cardiomyocytes, and myeloid leukocytes was downregulated after stimulation of TLR. CAP attenuated the expression of pro-inflammatory cytokines, such as IL-6, by limiting the phosphorylation of inhibitor of kappa B (IκB) kinase (Iκκ)-α and Iκκ-β in addition to inhibiting their NF-κB-dependent downstream signaling pathway. The presence of molecular affinity between CAP and Iκκ-α/Iκκ-β was a critical factor in disrupting the NF-κB pathway. CAP is thus an efficient adaptor molecule in inhibiting the NF-κB pathway (237).

## 21 Src kinase-associated phosphoprotein 2

The Src kinase-associated phosphoprotein (SKAP) proteins consist of SKAP1 [also referred to as SKAP55 (Src kinase-associated phosphoprotein of 55 kDa)] and its homolog SKAP2 [also referred to as SKAP-HOM (SKAP55-homolog) or SKAP-55R [SKAP-55-related]]. Both proteins have the same domain composition as a dimerization domain (DM), a pleckstrin homology domain (PH), and a C-terminal Src homology 3 domain (SH3). At the protein level, they are 44% identical, mainly in their PH and SH3 domains. Human SKAP1 contains three tyrosine-based signaling motifs at amino acid positions 219, 232, and 271 in the inter-domain, while human SKAP2 has only two motifs at amino acids 261 and 298 (238). SKAP2 is abundantly expressed in macrophages (239) as well as in T and B lymphocytes (238). It is a cytosolic adaptor protein found primarily in macrophages and plays an important role in cytoskeletal reorganization, macrophage migration, and chemotaxis.

An interesting study revealed the role of SKAP2 on macrophage podosome formation for the promotion of tumor invasion and metastasis. According to that study, peritoneal macrophages that are derived from SKAP2 null mice have lower invasive ability compared to those from the wild type. Also, injection of lung cancer cells in these mice leads to less lung metastasis, marked by lower macrophage infiltration in the tumor. Furthermore, when SKAP2 null macrophages were injected, the macrophage tumor infiltration and growth were reduced (240). It was observed that deficiency of SKAP2 in mice with colitis resulted in increased LPS-induced inflammation and tumorigenesis. Deletion of SKAP2 in colitis-induced mouse models demonstrated activation of the NF-κB pathway along with upregulation of cytokines such as TNF-α, CXCLs, and interleukins. Thus, consistent expression of SKAP2 is required to reduce inflammatory signal transduction in macrophages triggered by the uptake of exosomes from cancer cells. SKAP2 formed a complex with SHP-1 tyrosine phosphatase *via* association with the Sirpα transmembrane receptor. SKAP2 is also physically associated with the TIR domain of MyD88, TIRAP, and TRAM, adaptors of TLR4. SKAP2-mediated recruitment of the Sirpα/SHP-1 complex to TLR4 attenuated inflammatory responses, whereas direct interaction of SKAP2 with SHP-2 reduced activation of SHP-2. SHP-2 is required for efficient NF-κB activation and suppresses the TRAM/TRIF-IFN-β pathway; therefore, SKAP2-mediated SHP-2 inhibition affected two signaling axes of TLR4. Therefore, TLR4-NF-κB signaling is blocked and TLR4-IFN-β signaling is activated by SHP-1 and SHP-2 of SKAP2, effectively inhibiting inflammation-mediated tumorigenesis (239). In an *in vivo* study of atherosclerosis, SKAP2 was also found to be required for the expression of M2 polarization markers in addition to its athero-protective effects. Because SKAP2 binds to Sirpα, it is possible that Skap2 affects the interaction between CD47 and Sirpα (the “don’t eat me” signal) to promote efferocytosis by preventing antiphagocytosis. Although previous studies have found that blocking CD47 signaling to macrophages can increase efferocytosis and attenuate atherosclerosis, the mechanism remains to be explored (241). The connective role of Sirpa with SKAP2 was revealed in one more study, where it was shown that Sirpa is necessary for SKAP2 recruitment to engaged integrins and for regulating the downstream signaling of actin reorganization and cytoskeletal rearrangement, the critical steps for macrophage migration, chemotaxis, and phagocytosis (242).

## 22 Conclusion

Macrophages are central players in the innate immune response. Macrophage polarization, i.e., the dynamic adaptation of phenotypes, plays an important role in maintaining tissue homeostasis, tissue injury, and repair mechanisms by altering the tissue microenvironment. Adaptor molecules possess a number of domains that play a central role in the recruitment and transmission of inflammatory responses through intricate signaling cascades. They are also responsible for polarizing macrophages into two distinct states: the fierce M1 warriors, which play a pro-inflammatory role, and the calming M2 defenders, which are responsible for fighting inflammation. Several adaptor proteins reprogram macrophages to an anti-inflammatory M2 phenotype and thus control inflammation. Here, we have provided an overview of the role of adaptor molecules in impeding the inflammatory response and the associated signaling pathways, which are summarized in **Table 1**. Understanding the interactions of adaptor molecules in macrophage polarization is critical for elucidating the signaling pathways associated with inflammatory diseases and cancer and for developing novel therapeutic strategies. Based on the current findings mentioned above about adaptor proteins, we still need to understand more about their molecular mechanisms.

**Table 1:** Function of adaptor proteins in macrophage signaling pathway.

Adaptor protein	Function
SARM	Negative regulator of TLR3 and TLR4 (inhibits TRIF-dependent signaling).
DAP12	DAP12-deficient macrophages produced higher concentrations of inflammatory cytokines in response to a variety of pathogenic stimuli.
SOCS-1	Binds to TIRAP leading to U&PD and blocks the TLR4 and TLR2 signaling mainly.
IRAK-M	Negative regulator of TLR signaling. Prevents dissociation of IRAK-1/4 from MyD88. Hence, no formation of IRAK-TRAF6 complex reduced pro-inflammatory cytokine production.
DOK-3	Blocks LPS signaling in macrophages and reduces NF-κB activation.
IRF7	TIRAP binds with IRF7 and ceases its activation to block specifically IFN-β production.
NLRC5	Negative regulator of TLR signaling through IKK inhibition.
DAB2	Negative regulator of LPS stimulated TLR4 signaling.
Triad-3A	Promotes downregulation of two TIR domain-containing adapter proteins, TIRAP and TRIF.
CLIP170	Binds to TIRAP.
IRAK-1/4	When TIRAP binds to MYD88, then these act as signal-transducing relay molecules. However, when TIRAP directly interacts with IRAK-1/4, this leads to U&PD and finally leads to downregulation of inflammatory response mediated *via* TIRAP.
APS	Inhibit JAK signaling.
TOLLIP	Inhibitor of MyD88-dependent signaling cascade.
β-Arrestin	The interaction between β-arrestins and IκBα; TRAF6 inhibits NF-κB activity induced by inflammatory cytokines.
LnK	Inhibitor of TNF alpha-dependent pathway.
IRF4	Interacts with MyD88. Acts as negative regulator of TLR signaling. Competes with IRF5 for MyD88 interaction.
SLAP	Negative regulator of TCR signaling.
GPR108	Competes with TLR4 to bind to MyD88; in turn, the expression of GPR108 as an immune activator is restricted by TIRAP.
CAP	Inhibitor of NF-κB pathway.
SKAP2	Interacts with and activates SHP-2, therefore inhibiting NF-κB pathway, but activates TRIF/IFN-β pathway. Interacts with Sirpa and regulates macrophage phagocytosis.

SARM, Sterile α- and Armadillo motif-containing protein; DAP12, DNAX-activating protein of 12 kDa; SOCS-1, suppressor of cytokine signaling-1; IRAK-M, interleukin-1 receptor associated kinase-M; DOK-3, downstream of kinase 3; IRF7, interferon regulatory factor 7; NLRC5, NOD-like receptor family caspase recruitment domain family domain containing 5; DAB2, Disabled-2; CLIP170, cytoplasmic linker protein 170; IRAK-1/4, interleukin-1 receptor-associated kinase 1/4; APS, Astragalus polysaccharide; TOLLIP, Toll-interacting protein; IRF4, interferon regulatory factor 4; SLAP, Src-like adaptor protein; GPR108, G protein-coupled receptor 108; CAP, adaptor protein c-Cbl-associated protein; SKAP2, Src kinase-associated phosphoprotein 2.

Blocking or inhibiting adaptor proteins can shed more light on their role in regulating macrophage activation. There are certain pharmacological agents that act on adaptor molecules and are currently used in clinical trials as agonists of the adaptor molecule, playing an essential role in therapeutic purposes. For example, celastrol is one such drug that enhances the SARM expression and helps in decreasing the effect of incision-associated inflammation (243). Resveratrol is a drug that enhances SOCS-1 expression and helps in the inhibition of microglial activation (244). Pregnenolone is a drug that enhances CLIP170 expression during the degradation of TIRAP and TLR4 suppression (140). Glucocorticoids are drugs that suppress inflammation *via* the upregulation of IRAK-M (245). Similarly, other adaptor molecules can be targeted with pharmacological inhibitors to dampen not only the inflammatory response but also cancers that are closely associated with inflammation. Researchers are exploring the potential role of inflammation in many aspects of cancer, including the spread of the disease within the body and the resistance of tumors to treatment. Moreover, investigating adaptor functions in macrophage crosstalk with other immune cells and tumor cells would provide the tools to better understand cancer progression and design more precise therapeutic interventions.

## Author contributions

MB: Conceptualization, Funding acquisition, Writing – original draft, Writing – review & editing. SpB: Writing – review & editing. ShB: Writing – review & editing. AW: Writing – review & editing. NH: Writing – review & editing. RA: Writing – review & editing. RK: Writing – review & editing. RSh: Writing – review & editing. SS: Writing – review & editing. RSa: Writing – review & editing, Conceptualization, Funding acquisition, Writing – original draft.
